# Supplementary figures and images for: Molecular and electrophysiological features of GABAergic neurons in the dentate gyrus reveal limited homology with cortical interneurons
Source: PLoS One. 2022 Jul 8;17(7):e0270981. doi: 10.1371/journal.pone.0270981 (PMC9269967; doi:10.1371/journal.pone.0270981)

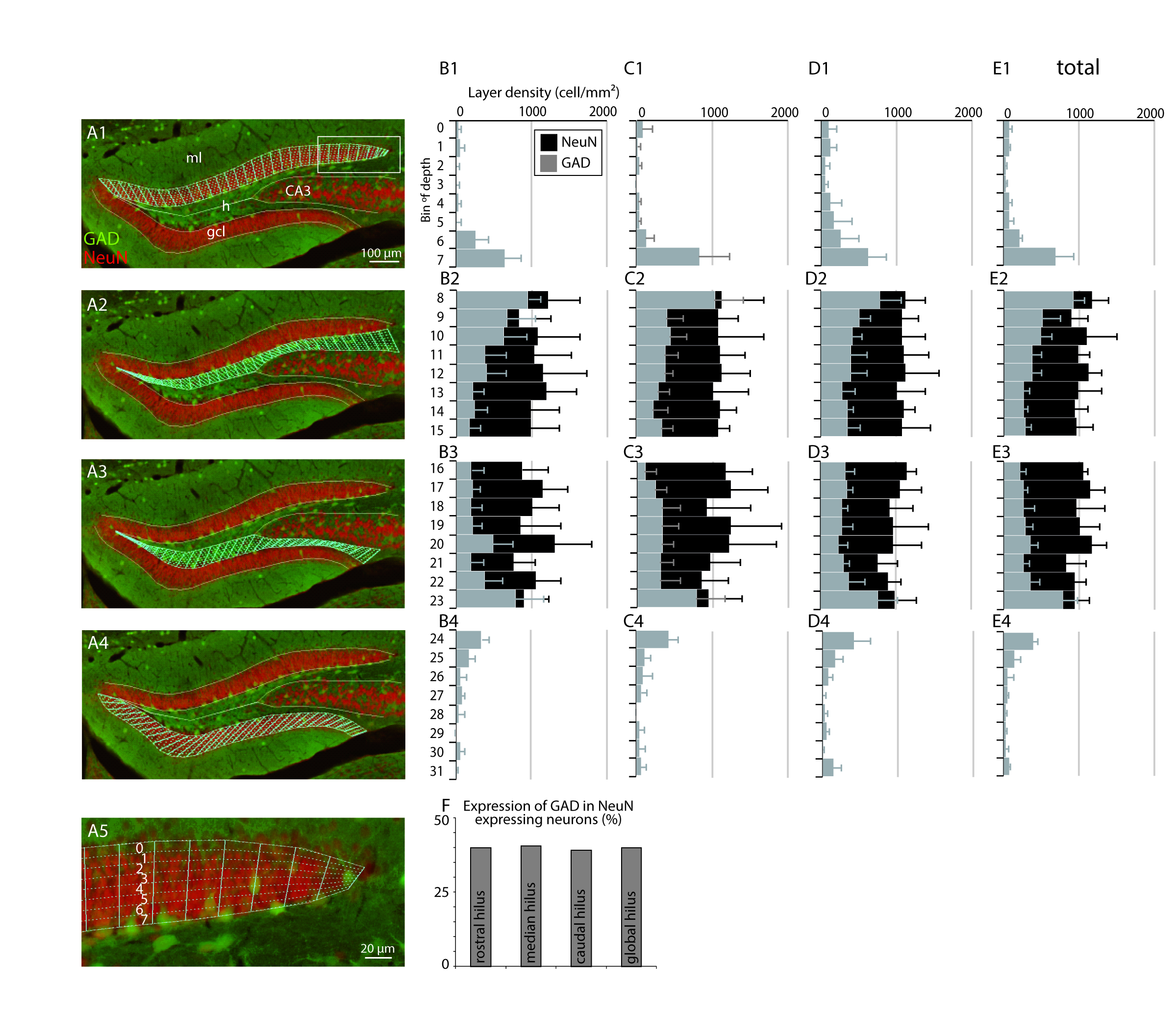

Supplement: S1 Fig — A1-A4: Representative photomicrograph of GFP and NeuN expression in rostral sections of the dentate gyrus of GAD67-GFP knock-in mice. Each of the following layers was divided into 8 bins using a semiautomated procedure (Materials and methods): A1: granular cell layer outer blade (bin 0 to 7), A2: hilar outer half (bin 8 to 15), A3: hilar inner half (bin 16 to 23) and A4: granular cell layer inner blade (bin 24 to 31). The delineated area in A1 is enlarged in A5. B-E: Histograms of the densities of GFP (grey) and NeuN (black) expressing cells in A: rostral, B: median and C: caudal slices and in E: all analyzed areas, for 1: bin 0 to 7, 2: bin 8 to 15, 3: bin 16 to 23 and 4: bin 24 to 31. F: Expression of GAD in NeuN expressing cells. (n = 7 mice, error bars: sem, gcl: granular cell layer, h: hilus, ml: molecular layer). (TIF) [file pone.0270981.s001.tif]

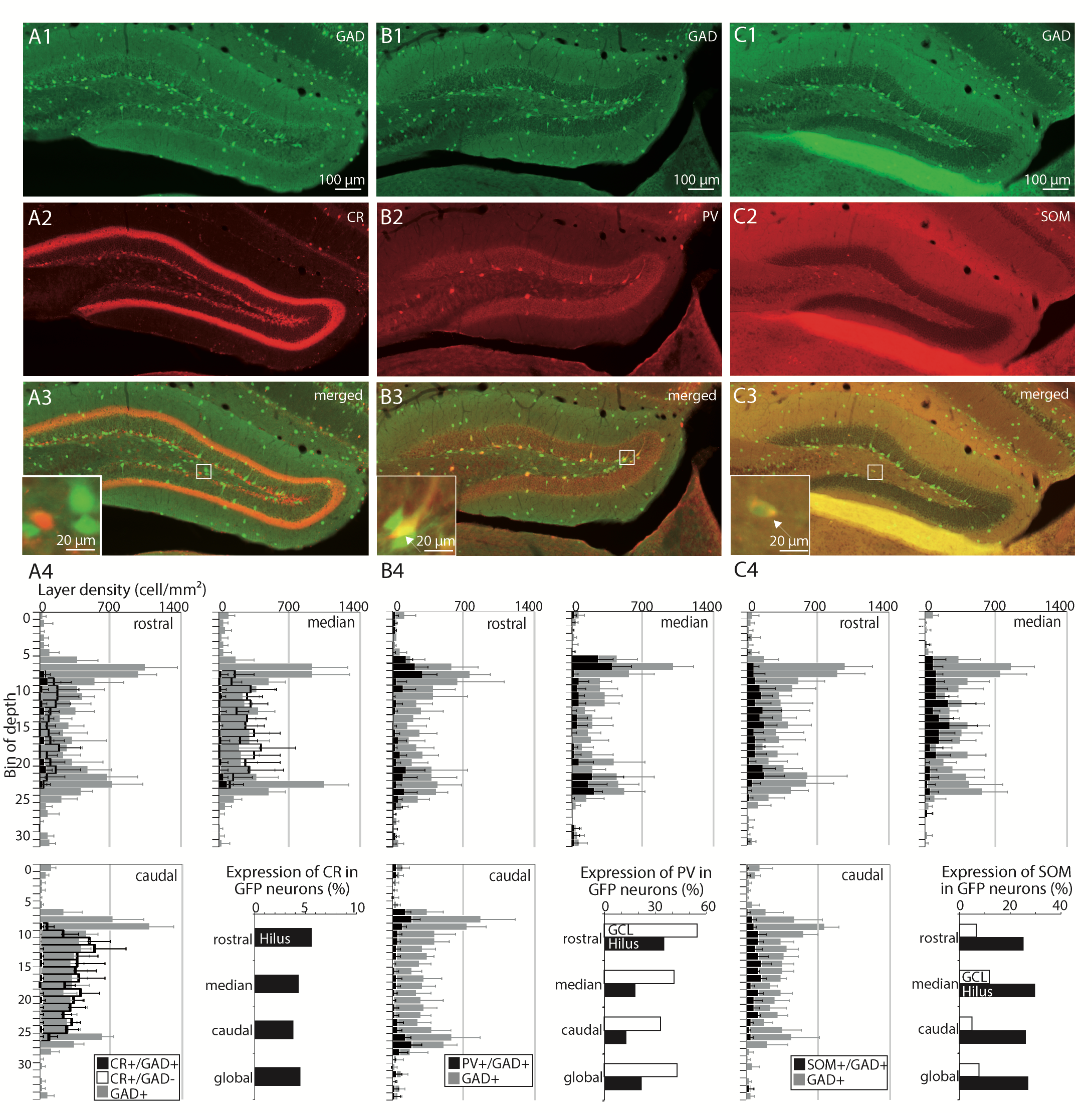

Supplement: S2 Fig — A1-A3: CR immunostaining in the dentate gyrus of a GAD67 GFP knock-in mouse. In A3, the delineated area is enlarged in the inset. Examples of GAD expressing neurons labeled with CR are pointed out with arrows. A4: Densities of GFP expressing cells (grey), CR expressing cells (black outline) and CR positive GFP expressing cells (black) in rostral, median and caudal slices. CR positive cells were not counted in bin 0 to 7 and 24 to 31. The histogram in the bottom right represents the percentage of expression of CR in GAD67 expressing cells in the hilus. B1-B3: PV immunostaining in the dentate gyrus of a GAD67-GFP knock-in mouse, the delineated area is enlarged in the inset in B3. Examples of GAD-expressing neurons labeled with PV are pointed out with arrows. B4: Histograms representing the densities of GFP (grey) and PV (black) expressing cells in bin 0 to 31, in rostral, median and caudal slices. The histogram at the bottom right represents the percentage of expression of PV in GAD expressing cells in the hilus (black) and granular cell layer (white). C1-C4. As described in B1- B4 for SOM. (n = 7 mice/marker, error bars: sem). (TIF) [file pone.0270981.s002.tif]

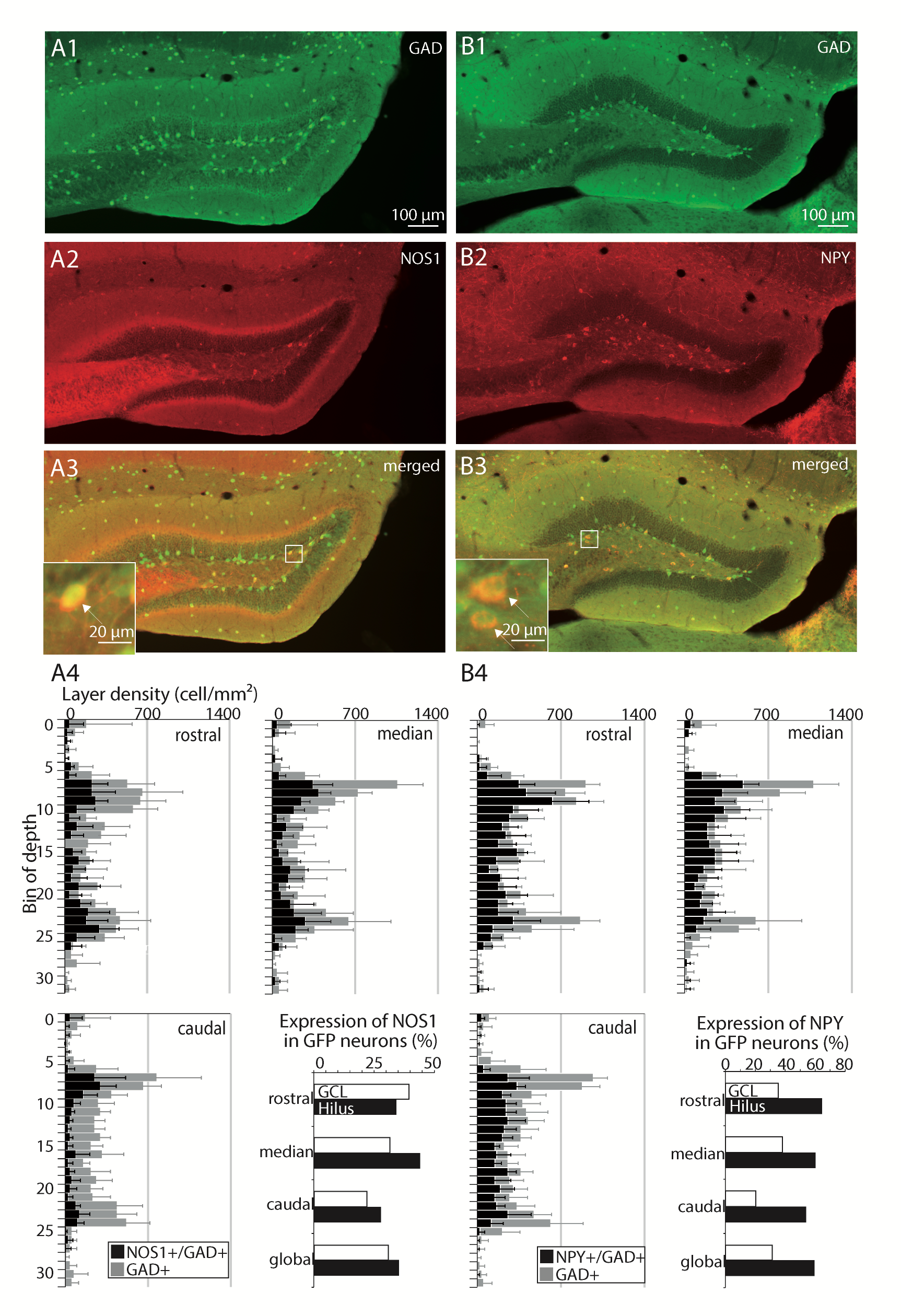

Supplement: S3 Fig — A1-A3: NOS-1 immunostaining in the dentate gyrus of a GAD67-GFP knock-in mouse. In A3, the delineated area is enlarged in the inset. Examples of GAD67 expressing neurons labelled with NOS1 are pointed out with arrows. A4: Histograms represent the densities of GFP (grey) and NOS1 (black) expressing cells in bin 0 to 31, in rostral, median and caudal slices. The histogram at the bottom right represents the percentage of expression of NOS1 in GAD expressing cells in the hilus (black) and granular cell layer (white). B1-B4: As described in A1-A4 for NPY. (n = 7 mice/marker, error bars: sem). (TIF) [file pone.0270981.s003.tif]

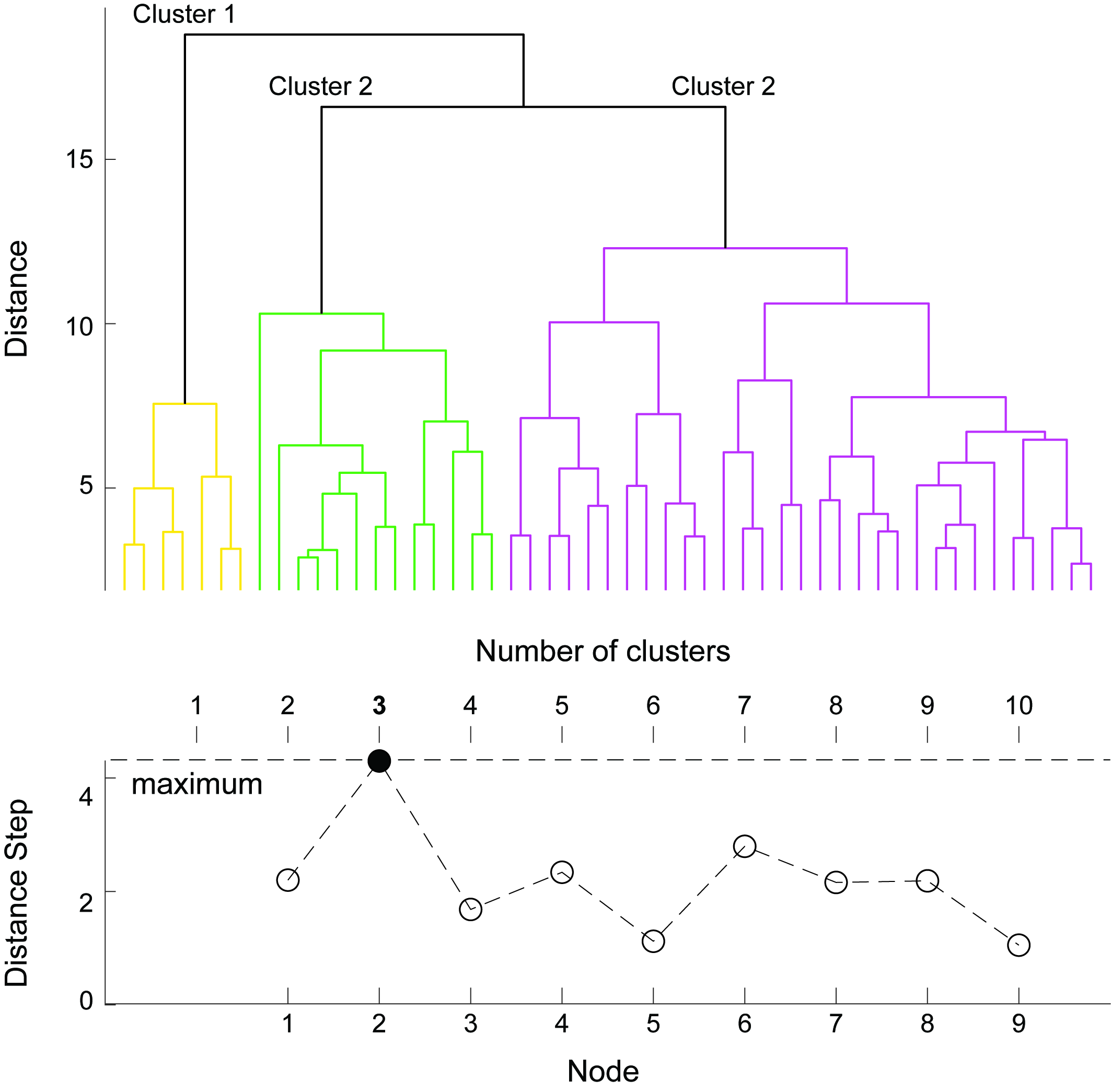

Supplement: S4 Fig — Top: Dendrogram of aggregation applying Ward’s unsupervised clustering to 51 hilar GABAergic neurons. 16 electrophysiological and 8 molecular parameters were used. The x axis represents individual cells and the y axis the distance of aggregation. Bottom: Distance to the closest downstream node after the first 9 nodes of the dendrogram. Distance is maximal after node 2 segregating 3 clusters. (TIF) [file pone.0270981.s004.tif]

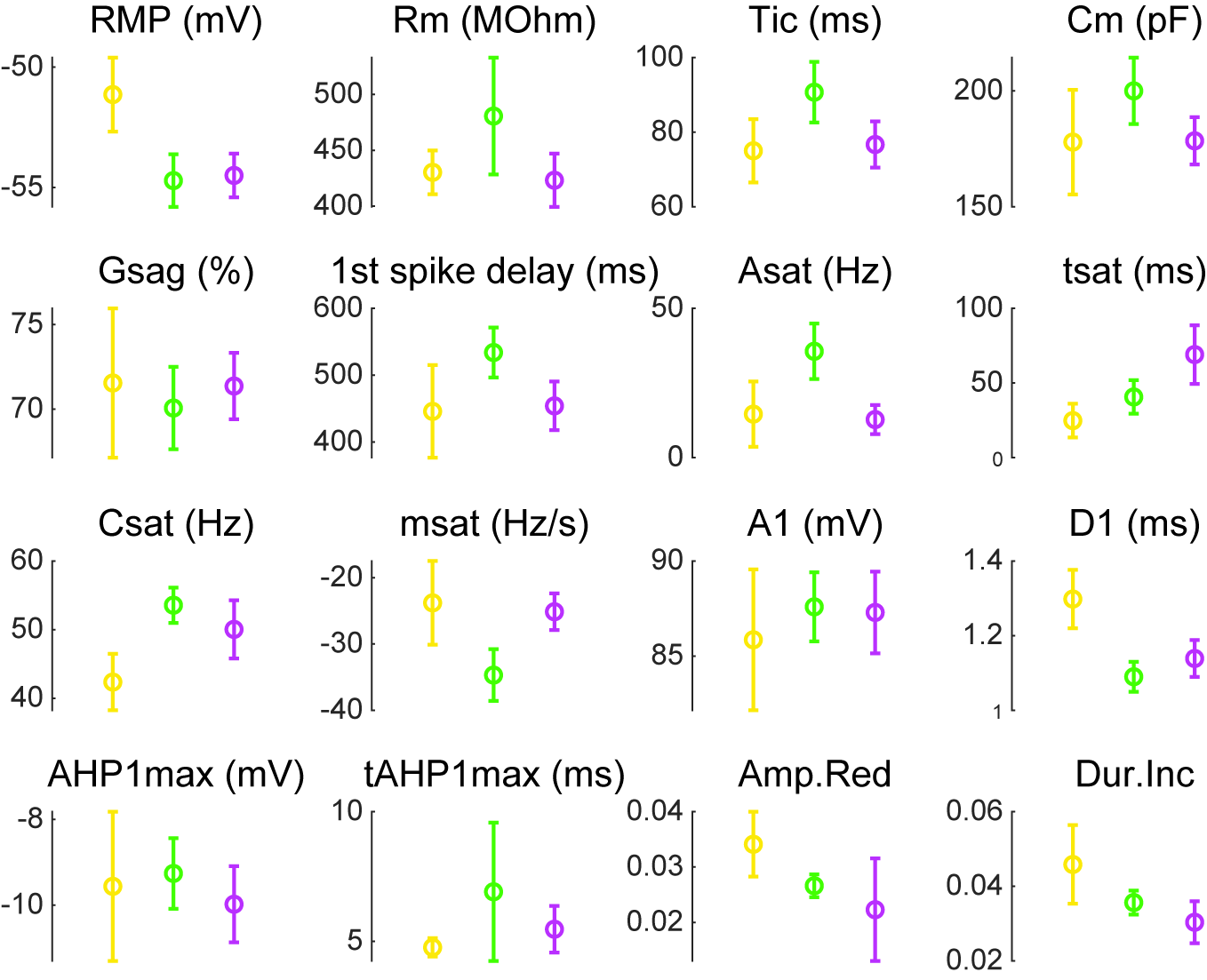

Supplement: S5 Fig — Values are represented as mean +/- sem. for each parameter and each cluster (yellow: cluster 1; green: cluster 2; purple: cluster 3; RMP: Resting membrane potential; Rm: Input resistance; Tic: Time constant of membrane capacitance; Cm: Membrane capacitance; Gsag: Rectification of hyperpolarization; 1st spike delay: delay to first spike from the onset of current injection at rheobase; Asat: Amplitude of adaptation near saturation; tsat: Time constant of adaptation near saturation; msat: Slope of adaptation near saturation; Csat: intersect of adaptation near saturation; A1: Amplitude of the first spike; D1: Duration of the first spike; AHP1max: Amplitude of after hyperpolarization potential; tAHP1max: Time of maximum after hyperpolarization potential; Amp. Red.: Amplitude reduction between the first and second spike at rheobase; Dur. Inc.: Duration increase between the first and second spike at rheobase; Material and methods). (TIF) [file pone.0270981.s005.tif]

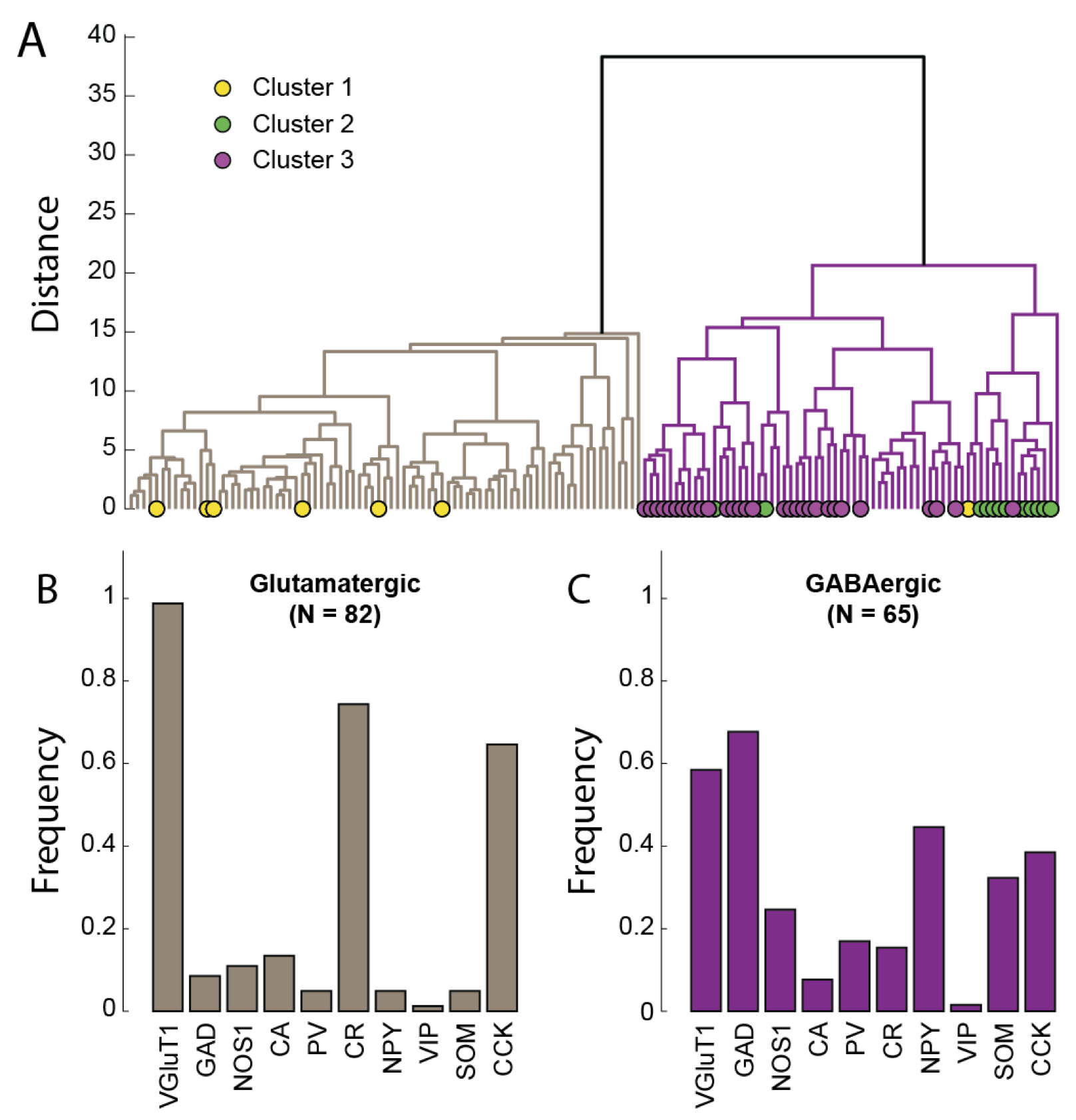

Supplement: S6 Fig — A. Ward’s unsupervised clustering applied to 147 hilar neurons based on their electrophysiological and molecular properties characterized with single cell RT-PCR. The same 16 electrophysiological and 8 molecular parameters were used as previously (Fig 2). The analysis disclosed 2 main branches. Colored circles represent GABAergic neurons clustered as in Fig 2. (yellow: cluster 1; green: cluster 2; purple: cluster 3). Most cluster 1 neurons are grouped in Branch 1, whereas all neurons from clusters 2 and 3 are assigned to branch 2. B. Histogram showing the expression of molecular markers in branch 1 (left; grey) and branch 2 neurons (right; purple). Branch 1 mostly comprises glutamatergic neurons. (TIF) [file pone.0270981.s006.tif]

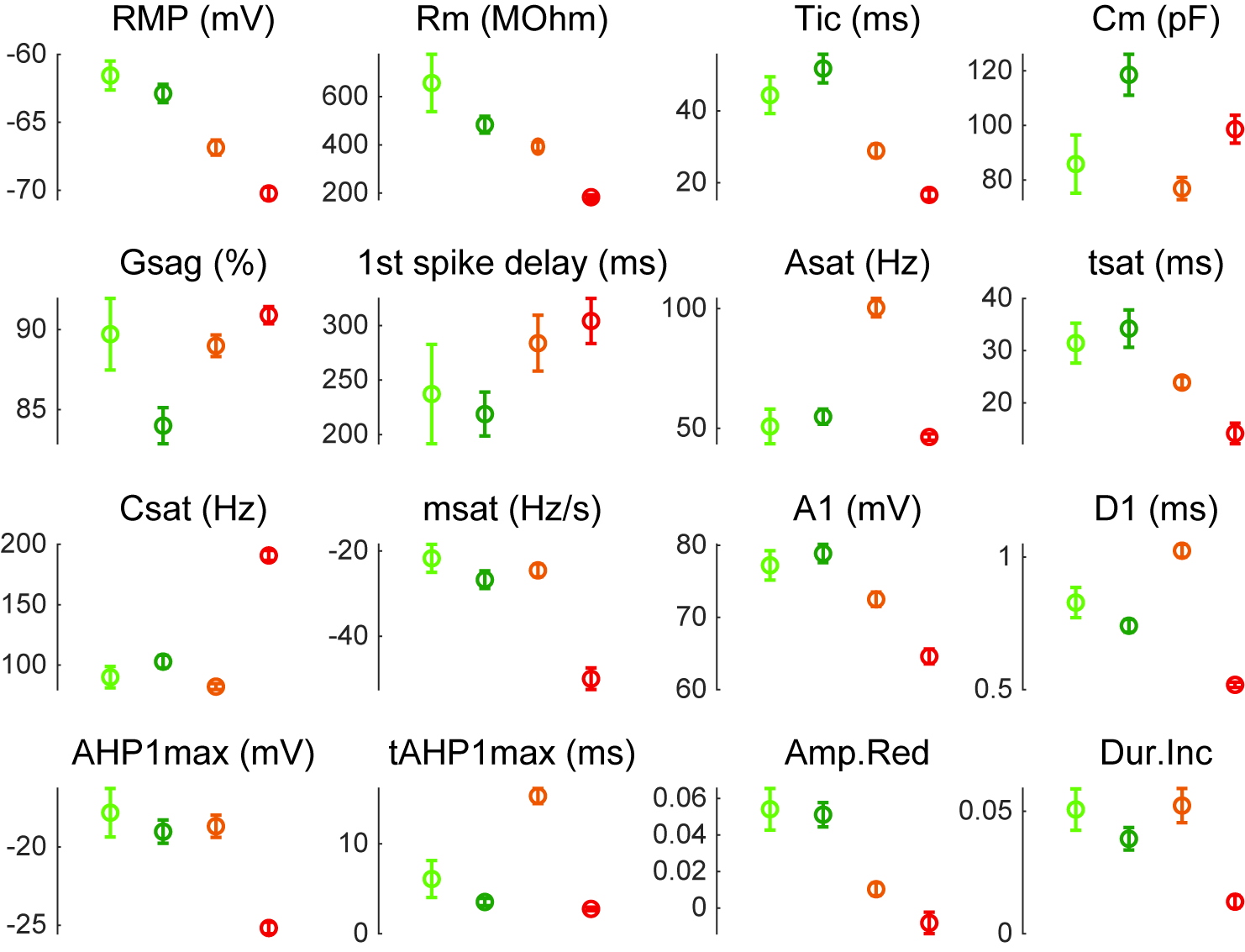

Supplement: S7 Fig — Values are represented as mean +/- sem. for each parameter and cluster (yellow: cluster 1; green: cluster 2; purple: cluster 3; RMP: Resting membrane potential; Rm: Input resistance; Tic: Time constant of membrane capacitance; Cm: Membrane capacitance; Gsag: Rectification of hyperpolarization; 1st spike delay: delay to first spike from the onset of current injection at rheobase; Asat: Amplitude of adaptation near saturation; tsat: Time constant of adaptation near saturation; msat: Slope of adaptation near saturation; Csat: intersect of adaptation near saturation; A1: Amplitude of the first spike; D1: Duration of the first spike; AHP1max: Amplitude of after hyperpolarization potential; tAHP1max: Time of maximum of after hyperpolarization potential; Amp. Red.: Amplitude reduction between the first and second spike at rheobase; Dur. Inc.: Duration increase between the first and second spike at rheobase; Material and methods). (TIF) [file pone.0270981.s007.tif]

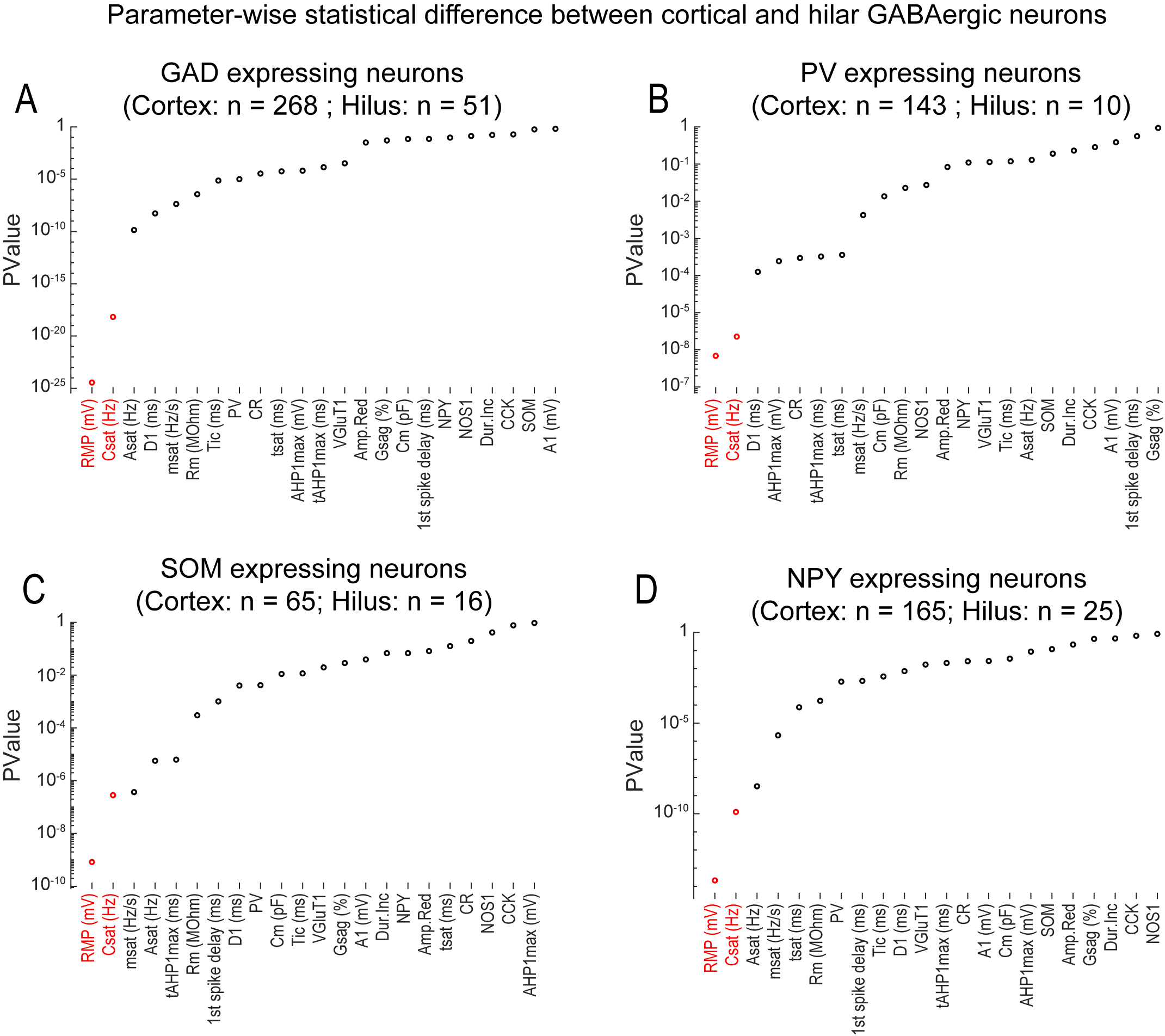

Supplement: S8 Fig — P-values of the statistical test of the difference between hilar and cortical GABAergic interneurons (Mann-Whitney rank-sum test). Values are sorted in ascending order. Different sets of cortical and hilar neurons were used based on markers expression. Marker used were A. GAD (i.e. full samples), B. PV, C. SOM and D. NPY. RMP and Csat (red) showed highly significant differences indicating a potential sample wise bias. (TIF) [file pone.0270981.s008.tif]

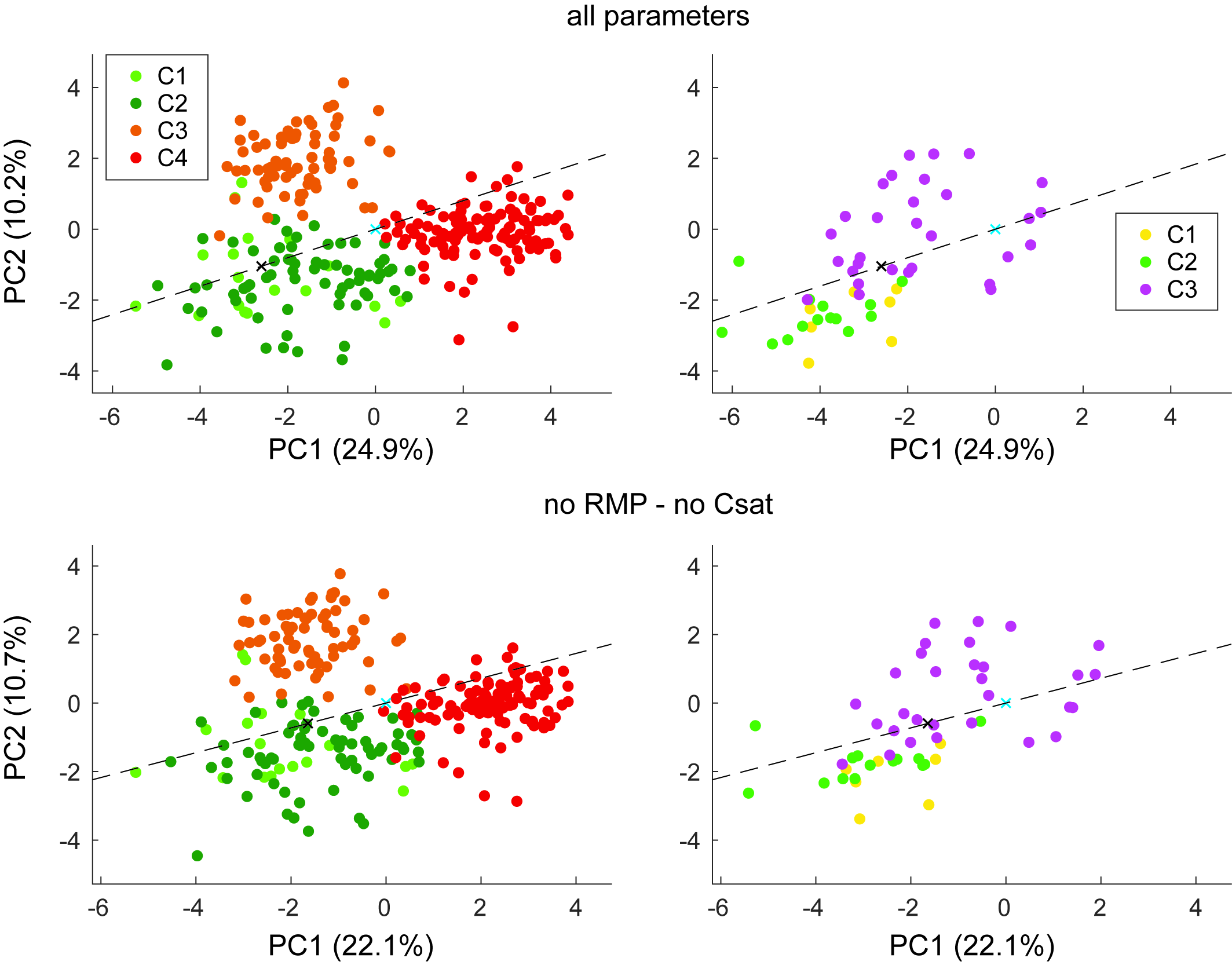

Supplement: S9 Fig — Clusters of cortical (left) and hilar GABAergic neurons (right) projected on the two first principal components of the cortical population using the full parameter set (top) or a parameter set excluding RMP and Csat (bottom), which might be subjected to population wise biases. Percentage in the x and y axes labels indicate each PC’s explained variance Crosses represent population centroids (black: cortex, blue: hilus) and dashed lines represent centroid-centroid axes. Removing RMP and Csat minimally affects the spread of cortical and hilar neurons and the positions of centroids along the 2 first principal components. (TIF) [file pone.0270981.s009.tif]
