# Supplementary material for: Molecular and electrophysiological features of GABAergic neurons in the dentate gyrus reveal limited homology with cortical interneurons
Source: PLoS One. 2022 Jul 8;17(7):e0270981. doi: 10.1371/journal.pone.0270981 (PMC9269967; doi:10.1371/journal.pone.0270981)
Supplement: Supplementary file 2 [file pone.0270981.s010.docx]

	VGluT1	NOS1	PV	CR	NPY	SOM	CCK
VGluT1		0.090969	0.398025	0.009823	0.784191	0.472021	0.604773
NOS1	0.090969		0.542802	0.894349	0.398025	0.50551	0.522903
PV	0.398025	0.542802		0.144061	0.090969	0.50551	0.337904
CR	0.009823	0.894349	0.144061		1	0.734586	0.464214
NPY	0.784191	0.398025	0.090969	1		0.150323	0.604773
SOM	0.949823	0.889673	0.889673	0.373488	0.892731		0.727628
CCK	0.604773	0.522903	0.337904	0.464214	0.604773	0.276895	
