# Supplementary material for: Molecular and electrophysiological features of GABAergic neurons in the dentate gyrus reveal limited homology with cortical interneurons
Source: PLoS One. 2022 Jul 8;17(7):e0270981. doi: 10.1371/journal.pone.0270981 (PMC9269967; doi:10.1371/journal.pone.0270981)
Supplement: Attachment — Submitted filename: Response to Reviewers.docx [file pone.0270981.s012.docx]

Dear Dr Biagini

Thank you very much for your renewed support of the publication of our manuscript in PLOS ONE. Please find here, our response to the additional suggestions raised by reviewer #2.

In order to make the tracking of corrections as clear as possible, we have highlighted in the revised manuscript in yellow the text that has been added, and crossed out the text that has been removed and highlighted in green. As a result, the page numbers that are referenced below take into account this follow-up of the corrections

**Reviewer #2:**

The authors have addressed the points raised. I still have a few minor requests.
1) The percentage of variance explained by PC1 and PC2 was not added to the graph in Fig. 2D. Unless there is a specific reason for this, it would be nice to have them.

We are grateful to the reviewer to point this out. The percentage of the variance of our hilar interneuron sample explained by PC1 and PC2 has been added to the graphs in Figure 2D. As can be seen there, and further clarified in the results (P14, L5-6) PC1 and PC2 accounted for 19.2% and 18% of the variance, respectively.

2) I appreciated the clarification that the PCA plots (in Fig. 4C, for example) are just a graphical representation and do not relate to a statistical analysis. To help the reader navigate this analysis, the figures/text could better represent this point. I suggest that the authors write the % explained by the PC only in the graph relating to the cortex (so, for ex. in Fig 4C, “PC Cortex (22.1%)” would have to be moved to the graph above). The graphs below would not have the % since it does not relate to the representation in the graph. If my understanding is correct, the analysis was done in two steps. First, the PCs (and the % variance explained) for cortical neurons were determined. Second, hilar neurons were projected on the parameter space of the Cortex PCs to visually inspect the superimposition of the two sets of dots. This clarification, although a little pedantic, would help a non-expert reader understand better.

We thank the reviewer for this excellent suggestion. Following the reviewers request, the percentage of variance is now only adjoined to the plots of our cortical sample in Figure 4 and 5. In addition, following the reviewer’s insight, we have reformulated our description of our PCA visualization in the results of the revised manuscript to better describe the two steps of the procedure (P18, L12-16). We have also specified in the corresponding legends the nature of the axes of the figures 2D (P15, L2-3), 4C (P18, L35-36) and 5B (P20, L37). We feel that this clarifies our analyses and make our manuscript more understandable.
